# Bacteria-driven nanosonosensitizer delivery system for enhanced breast cancer treatment through sonodynamic therapy-induced immunogenic cell death

**DOI:** 10.1186/s12951-024-02437-0

**Published:** 2024-04-12

**Authors:** Meng Du, Ting Wang, Wangrui Peng, Renjie Feng, MeeiChyn Goh, Zhiyi Chen

**Affiliations:** 1 Key Laboratory of Medical Imaging Precision Theranostics and Radiation Protection, College of Hunan Province, The Affiliated Changsha Central Hospital, Hengyang Medical School, University of South China, Changsha, Hunan, 410004 China; 2 Institute of Medical Imaging, Hengyang Medical School, University of South China, Hengyang, Hunan, 421001 China; 3 Medical Imaging Centre, The First Affiliated Hospital, Hengyang Medical School, University of South China, Hengyang, Hunan, 421001 China; 4 The Seventh Affiliated Hospital, Hengyang Medical School, University of South China (Hunan Provincial Veterans Administration Hospital), Changsha, Hunan 410118 China; 5 The Affiliated Changsha Central Hospital, Hengyang Medical School, University of South China, Changsha, Hunan 410004 China

**Keywords:** Sonodynamic therapy, Nanosonosensitizer, Bacteria, Tumor targeting, Cancer therapy

## Abstract

**Background:**

Sonodynamic therapy (SDT) has shown promise as a non-invasive cancer treatment due to its local effects and excellent tissue penetration. However, the limited accumulation of sonosensitizers at the tumor site hinders its therapeutic efficacy. Although nanosonosensitizers have improved local tumor accumulation through passive targeting via the enhanced permeability and retention effect (EPR), achieving sufficient accumulation and penetration into tumors remains challenging due to tumor heterogeneity and inaccurate targeting. Bacteria have become a promising biological carrier due to their unique characteristic of active targeting and deeper penetration into the tumor.

**Methods:**

In this study, we developed nanosonosensitizers consisting of sonosensitizer, hematoporphyrin monomethyl ether (HMME), and perfluoro-n-pentane (PFP) loaded poly (lactic-co-glycolic) acid (PLGA) nanodroplets (HPNDs). These HPNDs were covalently conjugated onto the surface of *Escherichia coli* Nissle 1917 (EcN) using carbodiimine chemistry. EcN acted as an active targeting micromotor for efficient transportation of the nanosonosensitizers to the tumor site in triple-negative breast cancer (TNBC) treatment. Under ultrasound cavitation, the HPNDs were disrupted, releasing HMME and facilitating its uptakes by cancer cells. This process induced reactive oxygen species (ROS)-mediated cell apoptosis and immunogenic cell death (ICD) in vitro and in vivo.

**Results:**

Our bacteria-driven nanosonosensitizer delivery system (HPNDs@EcN) achieved superior tumor localization of HMME in vivo compared to the group treated with only nanosonosensitizers. This enhanced local accumulation further improved the therapeutic effect of SDT induced-ICD therapeutic effect and inhibited tumor metastasis under ultrasound stimulation.

**Conclusions:**

Our research demonstrates the potential of this ultrasound-responsive bacteria-driven nanosonosensitizer delivery system for SDT in TNBC. The combination of targeted delivery using bacteria and nanosonosensitizer-based therapy holds promise for achieving improved treatment outcomes by enhancing local tumor accumulation and stimulating ICD.

**Graphical Abstract:**

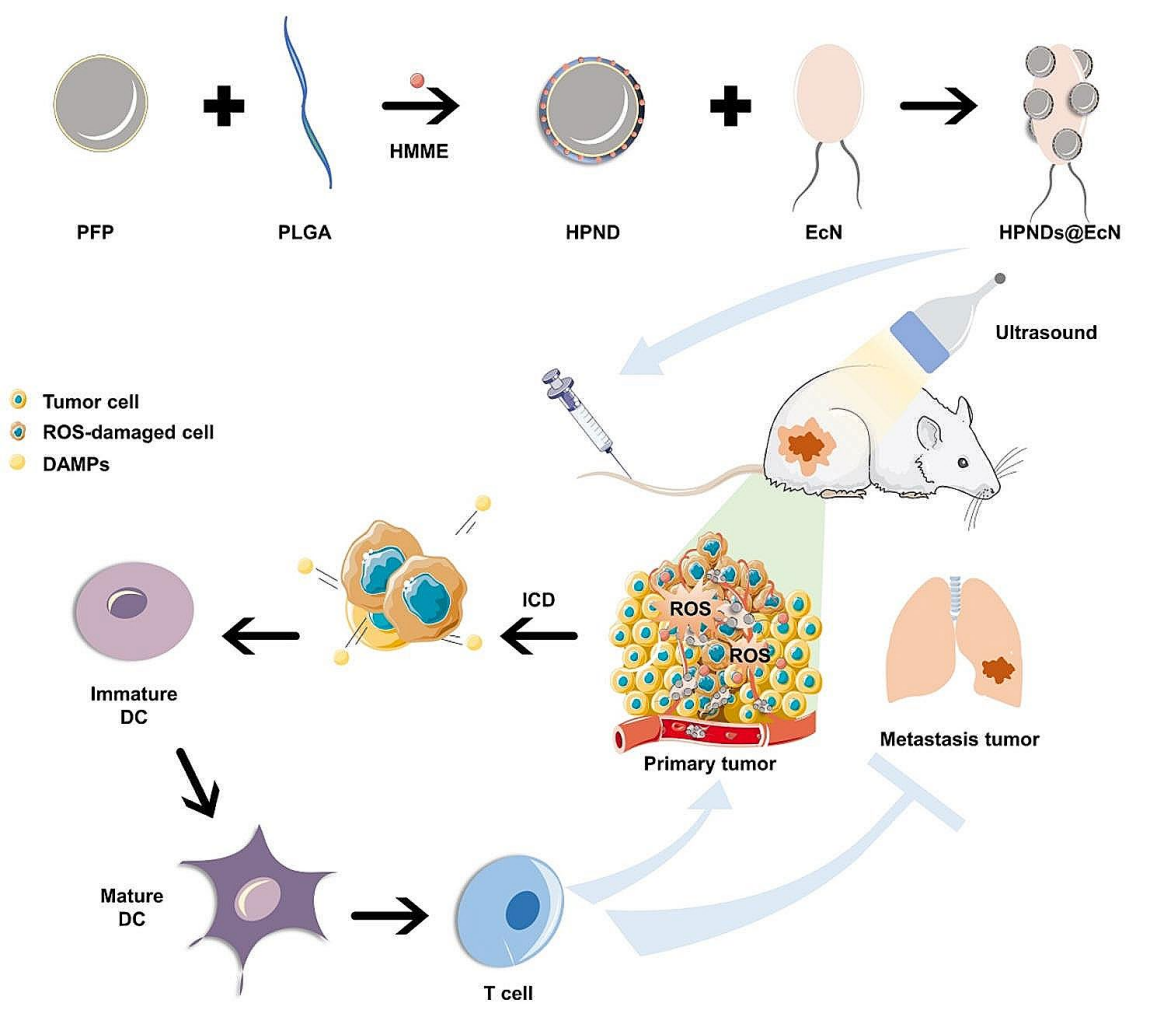

**Supplementary Information:**

The online version contains supplementary material available at 10.1186/s12951-024-02437-0.

## Introduction

Sonodynamic therapy (SDT) is an emerging and promising approach for the treatment of cancer by triggering immunogenic cell death (ICD) [1]. SDT-induced ICD utilizes low-intensity ultrasound (US) to activate sonosensitizers, which in turn generate reactive oxygen species (ROS) that induce apoptosis in cancer cells [2]. Subsequently, the activation of T lymphocytes occurs, leading to effective anti-tumor effects. SDT offers several advantages over photo-inspired therapies in tumor immunotherapy, including deeper tissue penetration ability, minimal side effects, and precise spatial and temporal selectivity [3–5]. Consequently, SDT is gaining significant attention as a non-invasive therapeutic modality and holds great potential in the field of cancer treatment [6].

The successful implementation of SDT heavily relies on achieving optimal local accumulation of sonosensitizers within the tumor microenvironment [7], thereby enhancing therapeutic efficiency. However, small molecule sonosensitizers lack selectivity towards tumor tissues, resulting in non-specific distribution and limited tumor accumulation [8]. Additionally, rapid clearance from the bloodstream and rapid metabolism within the body further limit their circulation time and reduce the opportunity for efficient tumor accumulation. To address this limitation, researchers have turned their attention to the development of nanoparticle-based sonosensitizers [9–11]. Nanoparticles have emerged as promising carriers for sonosenstizers, addressing the limitations of small molecule sonosensitizers [2]. By encapsulating or conjugating sonosensitizers onto nanoparticles [12], their stability can be improved, leading to prolonged circulation and increased accumulation within tumors [13, 14].

Nanosonosensitizers have shown great promise in enhancing tumor targeting for SDT [5, 6, 15]. However, several limitations still need to be addressed to maximize their efficacy [16]. One significant limitation is the challenge of achieving sufficient accumulation and deep penetration of nanosonosensitizers into the tumor tissue, particularly in hypoxic regions, utilizing the enhanced permeability and retention effect (EPR) [17, 18]. The dense extracellular matrix and abnormal blood vessel structure within tumors hinder the efficient distribution of nanosonosensitizers, particularly in effectively treating the core regions of the tumor where optimal treatment is crucial. Furthermore, tumors exhibit significant heterogeneity in terms of blood flow, oxygenation levels, and receptor expression across different areas [19]. This heterogeneity creates a barrier to achieving uniform targeting and delivery of nanosonosensitizers to all tumor cells [17, 20]. Consequently, certain regions may receive inadequate concentrations of nanosonosensitizers, leading to reduced overall treatment efficacy. Overcoming these limitations is of utmost importance to optimize the effectiveness of nanosonosensitizers in SDT [21].

Bacteria-driven drug delivery systems have garnered significant attention due to their potential for enhancing therapeutic specificity and efficacy in cancer treatment through active targeting [22, 23]. This innovative approach capitalizes on the inherent tropism of bacteria towards hypoxic regions and their ability to penetrate complex tumor microenvironments [24]. Various bacteria species such as *Bifidobacterium* [25], *Clostridium*, *Salmonella* Typhimurium [26], and *Escherichia coli* exhibit a preference for colonizing hypoxic, eutrophic, and immunosuppressive tumor tissues [27]. Among them, *Escherichia coli* Nissle 1917 (EcN), a biocompatible facultative anaerobic bacterium, has been identified as an ideal biological carrier with excellent tumor-targeting capabilities in cancer treatment [28]. EcN exhibits precise tumor-targeting capabilities, allowing for the delivery of therapeutic agents directly to the tumor site [29]. Therefore, the use of EcN as a biological carrier holds promise in overcoming the limitations of nanosonosensitizers in terms of efficient targeting, delivery, and accumulation in the tumor tissue. The integration of nanosonosensitizers with EcN can improve the therapeutic effect by enabling targeted delivery and local accumulation of drugs or therapeutic agents [30].

In our previous study, we fabricated a bacteria-driven drug delivery system using *E. coli* as a biological carrier for targeted drug delivery. Doxorubicin (DOX) and PFP-containing PLGA nanodroplets (DOX-PFP-PLGA) were attached to the *E. coli* [31]. Under ultrasound stimulation, the PFP in the nanodroplets underwent a phase transition from liquid to gas due to cavitation. This led to the disruption of the droplets and the release of DOX. This system enables DOX targeted delivery and controlled release at the tumor site through ultrasound stimulation. Inspired by this concept, we have developed a novel bacteria-driven nanosonosensitizer delivery system called HPNDs@EcN. This system is composed of hematoporphyrin monomethyl ether (HMME) [32], which serves as the porphyrins-based sonosensitizer, and PFP-loaded PLGA nanodroplets (HPNDs). These HPNDs are chemically conjugated onto the surface of EcN for targeted delivery in the context of SDT for triple-negative breast cancer (TNBC). The HPNDs@EcN system effectively retains the viability of EcN micromotors while preserving the therapeutic properties of HPNDs. In this study, we demonstrated the efficacy of SDT-induced local tumor cell death and inhibition of tumor metastasis in a TNBC mouse model under ultrasound stimulation. The utilization of this bacteria-driven nanosonosensitizer delivery system leveraging EcN enabled efficient delivery of HMME to the tumor site, subsequently leading to the successful induction of SDT-induced ICD. Overall, this bacteria-driven nanosonosensitier delivery system utilizing EcN demonstrated efficient delivery and accumulation of HMME to the tumor site and the potential for SDT-induced ICD.

## Materials and methods

### Materials

Poly (lactic-co-glycolic acid) decorated with -COOH (PLGA-COOH) was purchased from Shanghai Aladdin Biochemical Technology Co., Ltd (China). Perfluoro-n-pentane (PFP) was purchased from Strem Chemicals, Inc. Hematoporphyrin monomethyl ether (HMME) was purchased from Dibo Biology Technology Co., Ltd (China). Polyvinyl alcohol (PVA), CHCl_3_, 2-morpholinoethanesulfonic acid (MES), 1-ethyl-3-(3-dimethylaminopropyl)-carbodiimide (EDC), and Sulfo-NHS were purchased from Sigma-Aldrich.

### Preparation of HPNDs and HPNDs@EcN

PLGA-COOH and HMME were dissolved in CHCl_3_ to obtain a 2% solution. PFP was added to the solution and sonicated, followed by the collection of the primary emulsion and the addition of 4% PVA. After another round of sonication, a second emulsion was obtained and the emulsion was added with 2% isopropanol solution, and then magnetically stirred for 3 h to volatilize the CHCl_3_. The nanosonosensitizers, termed HPNDs were collected by rinsing with ddH_2_O and centrifugation.

To prepare HPNDs@EcN, the HPNDs obtained from the above procedure were first dissolved in MES buffer (0.1 m, pH 5.6), and EDC was added to sulfonyl-NHS in a molar ratio 30: 30: 1. The mixture was incubated at room temperature for 2 h, followed by centrifugation and resuspension of the precipitate with PBS to obtain HPNDs with activated -COOH. Next, a culture of EcN was added to the activated HPNDs and incubated in a shaker at 37 ℃ for 12 h. The HPNDs@EcN complex was purified by centrifugation and the resulting pellet was resuspended with PBS for further experiments.

### Characterization of HPNDs and HPNDs@EcN

The particle size and zeta potential of HPNDs were determined by dynamic light scattering (DLS) analysis using the Malvern NANO ZS instrument. The UV-vis absorption spectra of each component of HPNDs@EcN were measured using a UV spectrophotometer. Flow cytometry (Thermo Fisher Scientific, USA) was used to assess the connectivity efficiency between HPNDs and EcN. Before the transmission electron microscope (TEM), HPNDs@EcN samples were fixed with glutaraldehyde. EcN and HPNDs@EcN were cultured on LB-Agar plates, and the viability of EcN was determined by counting the number of colonies (clonogenic number).

### Cell culture

Mouse triple-negative breast cancer cell line, 4T1, was purchased from ATCC (USA). The cells were cultured in Dulbecco’s modified Eagle medium (DMEM, Gibco) supplemented with 10% fetal bovine serum and 1% penicillin-streptomycin solution under standard conditions of 5% CO_2_ and 37 ℃.

### Establishment of ultrasound irradiation system and procedure

The ultrasound irradiation system used in this study was purchased from SXUltrasonic (Shenzhen, China), which contains a 1 MHz unfocused ultrasound probe. The ultrasound parameters used in the cell experiments were 1.4 W/cm^2^ with a duty cycle of 50% and an irradiation time of 1 min. For the animal experiments, the ultrasound irradiation parameters were set to 1.4 W/cm^2^ with a duty cycle of 50%, and the total irradiation time was 10 minutes.

### HMME controlled release and cell uptake assay

4T1 cells were seeded at a density of 1 × 10^6^ per well in 12-well plates and incubated at 37 ℃ in a 5% CO_2_ incubator for 24 h. Subsequently, HPNDs@EcN, both with and without ultrasound treatment, were added and co-incubated with 4T1 cells for 4 h. After co-incubation, the cells were washed with PBS containing 1% gentamicin and then stained with DAPI. The intracellular cell uptake of HMME (indicated by red fluorescence) was visualized using confocal microscopy. For the in vitro controlled release experiment of HMME, HPNDs@EcN were added to 24 well plates and subjected to ultrasound irradiation. The supernatant was collected after centrifugation to measure the HMME release using a UV spectrophotometer. The percentage of HMME released was calculated based on the standard curve of HMME with known concentrations.

### Detection of intracellular ROS generation

4T1 cells were seeded into 24-well plates at a density of 1 × 10^5^ cells per well and cultured for an additional 12 h to allow them to fully adhere to the walls. The cells were then divided into four groups: Control group, US group, HPNDs@EcN group, and HPNDs@EcN + US group. For each group, the cell medium was replaced either with a serum-free medium or a medium containing HPNDs@EcN. After 4 h co-incubation of HPNDs-EcN, the cells were treated with DCFH-DA for 30 min and then exposed to ultrasound. The ultrasound irradiation was performed with the following parameters: 1 MHz frequency, 1.4 W/cm^2^ intensity, 50% duty cycle, and 1 min duration. After washing with PBS containing 1% gentamicin and staining with DAPI for 10 minutes, the intracellular green fluorescence signal was visualized under confocal microscopy. The mean fluorescence intensity of each group was quantified using Image J software, analyzing at least 3 microscopic fields in one sample and across a minimum of 3 samples.

### In vitro antitumor effects of HPNDs@EcN

To assess the antitumor effects of HPNDs@EcN, CCK-8 assay was conducted. The cell preparation and ultrasound treatment were similar to those described above. Following ultrasound irradiation, the samples underwent further incubation for 3 h, after which they were washed with PBS containing 200 µg/mL gentamicin. A 10% CCK-8 solution was then added to each sample and further incubated for 1 h before measuring the absorbance. The absorbance of each sample at 450 nm was detected using a multimode microplate reader (Synergy H1, USA). Live-dead double staining using calcein acetoxymethyl ester (Calcein AM) and propidium iodide (PI) was also conducted to determine the cell viability.

### In vitro detection of CRT exposure and HMGB1 distribution

The cell preparation and ultrasound treatment were conducted in a manner similar to those described previously. After washing 3 times with PBS containing 200 µg/mL gentamicin, the cells were incubated for an additional 24 h. The CRT exposure and intracellular HMGB1 distribution were determined by immunofluorescence staining for both CRT and HMGB1. The cells were first incubated with rabbit anti-mouse CRT primary antibody and rabbit anti-mouse HMGB1 primary antibody (Beyotime, Shanghai, China) followed by incubation with Alexa Fluor 488-coupled sheep anti-rabbit secondary antibody. The cells were then visualized using confocal microscopy. The mean fluorescence intensity of each group was quantified using Image J software, analyzing at least 3 microscopic fields from one sample and a minimum of 3 samples in total.

### Preparation of animal models

Balb/c female mice (4–6 weeks, 15–20 g) were purchased from the Experimental Animal Center of Guangdong Province. A 100 µL DMEM containing 1 × 10^6^ 4T1 cells was implanted subcutaneously into the right hind leg of each mouse to establish an animal tumor model.

### Tumor targeting ability experiment

After administering DiR-labeled HPNDs@EcN, the distribution of fluorescence signals (ex/em = 780/ 845 nm) was observed at different time points (before administration, 12 h, 24 h, 48 h, and 72 h after administration) using a Fluorescence imaging system (PerkinElmer, USA). After 72 h of administration, the tumor-bearing mice were executed, and the organs, as well as tumor tissues, were obtained for fluorescence signal detection using the same imaging system. Additionally, the accumulation of EcN in different organs was detected by clone counting. At 24 h, 48 h, and 72 h after administering HPNDs@EcN, tumors and organs were obtained, and diluted grinds of each organ were cultured on LB-Agar plates for clone counting.

### In vivo antitumor effects of HPNDs@EcN

To examine the effect of HPNDs@EcN-based SDT, the optimal ultrasound irradiation parameters (1 MHz, 1.4 W/cm^2^, 50% duty cycle, and 10 min) determined from in vitro treatment screening were used for in vivo animal experiments. 4T1 tumor-bearing mice were randomly divided into 4 groups: control group (PBS treatment), US group, HPNDs@EcN group, and HPNDs@EcN + US group. These mice were injected with 200 µL of PBS and HPNDs@EcN (2 × 10^7^ CFU per mouse, equivalent concentration of HMME was 1 mg/mL) via tail vein on days 10, 15, and 20 post-tumor implantation. Ultrasound irradiation was then applied 72 h following each tail vein injection. The efficacy of HPNDs@EcN-based SDT in treating breast cancer was evaluated by continuously monitoring tumor volume and weight in mice. After treatment, tumor tissue was extracted for histological examination. H&E staining was performed to observe the histological changes in the tumors across the different groups. Furthermore, TUNEL and Ki67 staining were conducted to evaluate the tumor cells’ apoptosis and proliferation. Additionally, to evaluate the effect of HPNDs@EcN SDT in inhibiting tumor metastasis, the lungs of the treated mice were excised at the end of treatment, and the number of lung metastases was counted.

### In vivo ICD measurement, DCs maturation, and CD8 ^**+**^T cell infiltration

The expression of ICD markers, including CRT and HMGB1, was examined in tumor tissue by immunohistochemistry. The maturation of intratumoral DCs and the infiltration of CD8^+^ T cells were analyzed with a flow cytometer. Tumors were collected and digested with collagenase V to obtain single-cell suspensions. Subsequently, the cells were stained with CD80-APC, CD86-PE, and CD8-PE. Following staining, cells were washed with a cell staining buffer and sorted using a flow cytometer.

### Statistical analysis

All in vitro and in vivo experiments were conducted at least 3 times. The experiment data were statistically analyzed using GraphPad Prism. The one-way analysis of variance (ANOVA) was used for statistical analysis at 95% and 99% confidence levels, respectively. The term “ns” indicates no significance, *p* < 0.05 were considered statistically significant. * indicates *p* < 0.05, ** indicates *p* < 0.01, *** indicates *p* < 0.001, and **** indicates *p* < 0.0001.

## Results

### Preparation and characterization of HPNDs@EcN

**Fig. 1:**
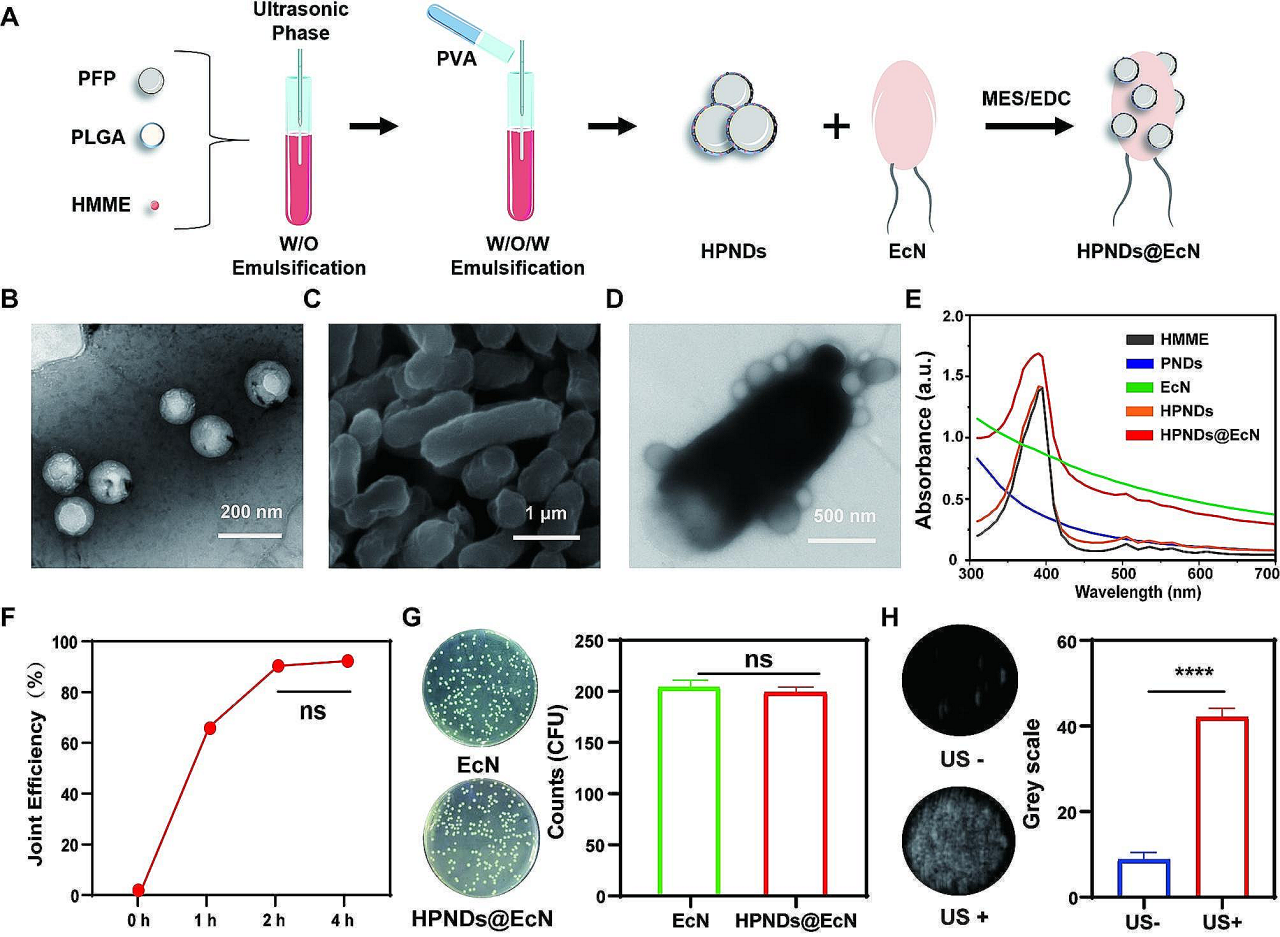
Preparation and characterization of HPNDs@EcN and corresponding functional verification. (**A**) Schematic diagram of HPNDs@EcN construction. (**B**) HPNDs observed by TEM. (**C**) EcN observed by SEM. (**D**) HPNDs@EcN observed by TEM. (**E**) UV-vis spectra of PNDs: PFP containing nanodroplets without HMME; HMME: hematoporphyrin monomethyl ether; EcN: *Escherichia coli* Nissle 1917; HPNDs: HMME/PFP nanodroplets; HPNDs@EcN: HMME/PFP nanodroplets linked *Escherichia coli* Nissle 1917. (**F**) HPNDs@EcN connection percentage detected at different reaction times. (**G**) Bacterial activity before and after HPNDs@EcN linkage. (H) Ultrasound imaging of HPNDs@EcN before and after ultrasound irradiation and its quantitative analysis. (ns: no significance, **** *p* < 0.0001)

In this study, ultrasound-responsive HMME/PFP nanodroplets (HPNDs) were prepared by the double emulsification method. Transmission electron microscopy (TEM) revealed that HPNDs had a spherical morphology. According to dynamic light scattering (DLS) measurements, HPNDs had a particle size of about 200 nm and a zeta potential of approximately − 20 mV (Fig. 1B, S1A). It is noteworthy that, the size of HNPDs did not show any significant changes after undergoing the ultrasound treatment (Fig. S1B). This phenomenon may be attributed to only the edges of PLGA nanoparticles being damage by the gas pressure formed from PFP during its phase transition from liquid to gas under the influence of ultrasound [33]. Furthermore, the particle size and zeta potential of HPNDs remained stable throughout 1 week of continuous monitoring in phosphate buffer saline (PBS), indicating their stability (Fig. S1C and S1D).

To observe the morphology of HPNDs and EcN after linking, the morphology of HPNDs@EcN was observed by electron microscopy and fluorescence microscopy. Under scanning electron microscopy (SEM), the probiotic EcN had a regular morphology with a rod shape (Fig. 1C). HPNDs@EcN had multiple HPNDs attached to the surface of EcN under TEM observation. The morphology of HPNDs@EcN was further observed by fluorescence microscopy. Since HMME can self-fluoresce in red, EcN that has been bound to HMME-loaded nanodroplets (HPNDs@EcN) was visible in red fluorescence under fluorescence microscopy (Fig. S2). The UV-vis spectra of HPNDs, HPNDs@EcN showed that both of them had HMME-specific absorption peaks at 390 nm (Fig. 1E). These findings indicated that the nanodroplets containing HMME are successfully linked to the EcN. The linkage efficiencies were 67.2%, 91.2%, and 93.1% at 1 h, 2 h, and 4 h, respectively, suggesting that the linkage efficiency increased with the increase of linkage time and reached a plateau at 2 h (Fig. 1F). Therefore, the optimal linkage time for nanodroplets to EcN was determined to be 2 h. To confirm the activity of probiotic bacteria after linking with HPNDs, the number of colonies of EcN and HPNDs@EcN was measured by the bacterial plate coating method. The findings revealed that the number of colonies in the HPNDs@EcN group was comparable to that in the EcN group, indicating that HPNDs had no impact on bacterial activity (Fig. 1G).

To investigate the phase transition ability of HPNDs@EcN in vitro, the ultrasound signals of HPNDs@EcN before and after the ultrasound irradiation were observed using an in vitro ultrasound diagnostic instrument. HPNDs@EcN showed significant signal enhancement after in vitro ultrasound irradiation, and the difference was statistically significant (*p* < 0.0001) (Fig. 1H).

### In vitro anti-tumor effect of HPNDs@EcN

**Fig. 2:**
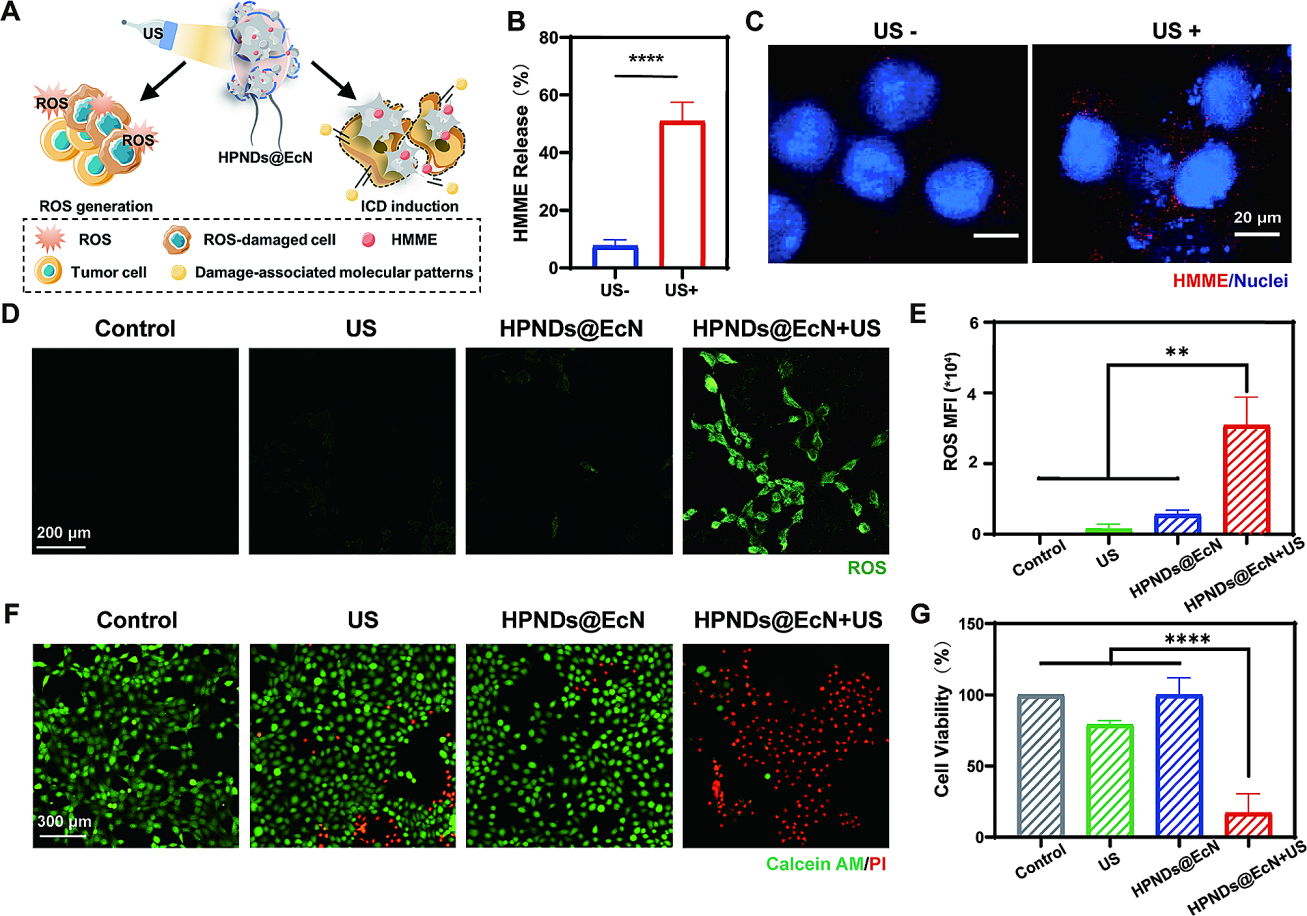
In vitro anti-tumor effect of HPNDs@EcN. (**A**) Schematic diagram of ultrasound mediated-SDT for tumor therapy. (**B**) HMME released from HPNDs@EcN with and without ultrasound treatment. (**C**) HMME intracellular uptake: Red: HMME; Blue: DAPI staining (scale bar: 20 μm). (**D**-**E**) Intracellular reactive oxygen production detection and quantification. (**F**-**G**) Cell cytotoxic qualitative and quantitative analysis with live dead double staining and CCK 8 assay. (** *p* < 0.01, **** *p* < 0.0001)

Due to the cavitation effects of ultrasound, HPNDs@EcN experience liquid-gas phase transition, which can result in the release of HMME. Therefore, it is not surprising that the amount of HMME released from nanodroplets after ultrasound irradiation is significantly higher than that without ultrasound irradiation (Fig. 2B). The cell uptake assay results showed that HMME red fluorescence signal detected in tumor cells co-incubated with HPNDs@EcN after ultrasound treatment was higher than the HPNDs@EcN without ultrasound treatment group (Fig. 2C), which is in line with the HMME released shown in Fig. 2B.

DCFH-DA (2,7-dichlorodihydrofluorescein diacetate) is a green fluorescent ROS probe that can freely enter and exit the cell membrane without fluorescence but can produce green fluorescent DCF (2,7-dichlorofluorescein) after being oxidized by intracellular ROS. As a result, the green fluorescent signal of DCF can reflect the level of intracellular ROS. In this study, DCFH-DA was used as a ROS detection probe to study the effect of ultrasound on initiating ROS production from HNPDs@EcN in 4T1 cells. Laser confocal fluorescence imaging revealed that the 4T1 cells in the HPNDs@EcN + US group had intracellular green fluorescence intensity that was significantly higher than that of the HPNDs@EcN, US, and control groups (Fig. 2D and E), indicating efficient ROS production from HMME under ultrasound irradiation. The live-dead cell staining results (Fig. 2F) and cytotoxicity experiments (Fig. 2G) further illustrated the obvious killing effect of HPNDs@EcN than the other groups on tumor cells after ultrasound irradiation.

### In vitro induction of ICD

**Fig. 3:**
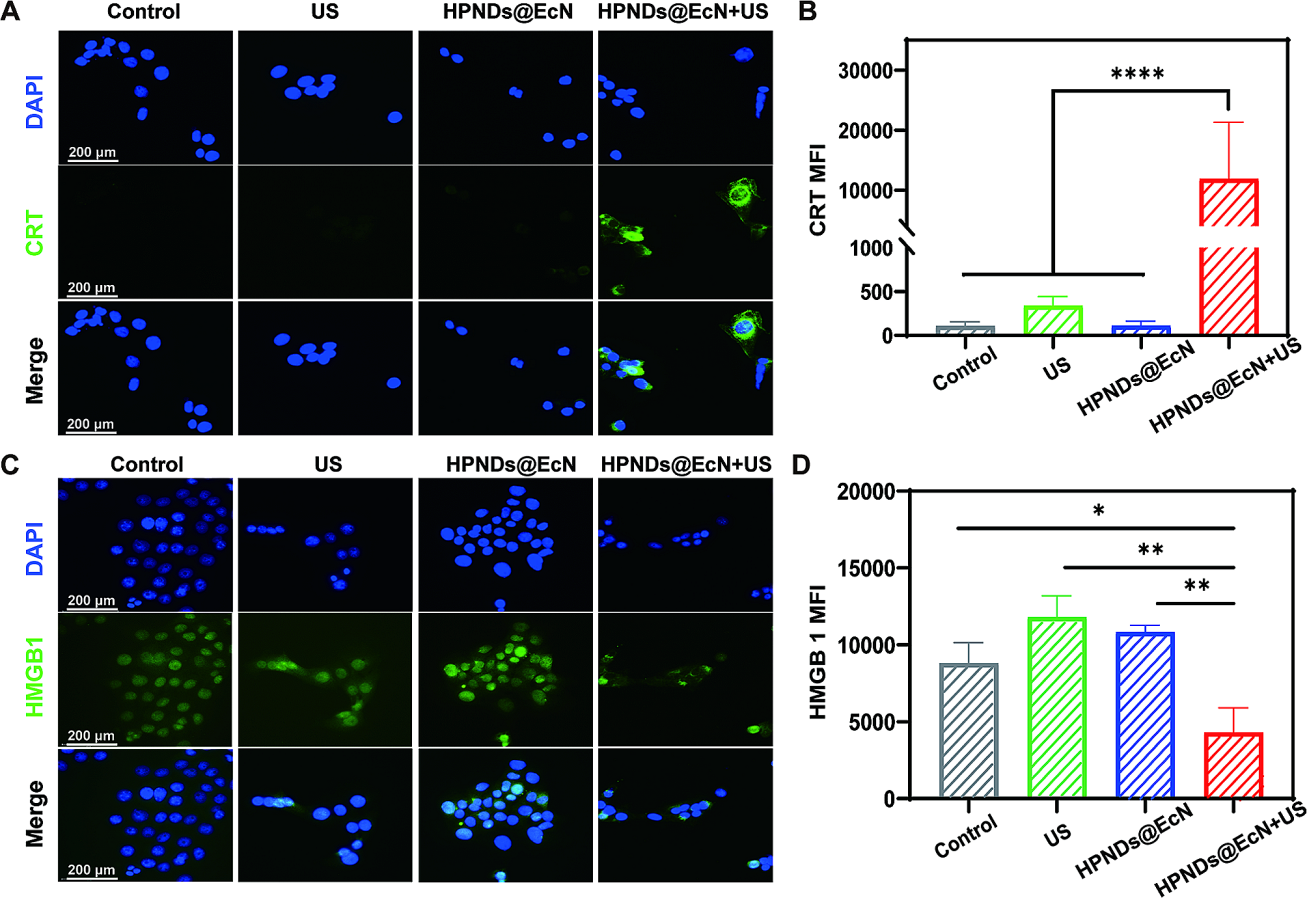
The efficiency of ICD induction in vitro. (**A**-**B**) CRT exposure level on the tumor cell surface and its quantification: Green: CRT; Blue: DAPI staining. (**C**-**D**) Intracellular HMGB1 secretion level and its quantification in the tumor cells: Green: HMGB1; Blue: DAPI staining. (* *p* < 0.05, ** *p* < 0.01, **** *p* < 0.0001)

CRT and HMGB1 are key biomarker molecules of immunogenic cell death. To detect the effect of SDT using HPNDs@EcN to induce immunogenic cell death in tumor cells, immunofluorescence staining was used to detect the exposure level of CRT on the cell membrane surface and the secretion level of HMGB1 in tumor cells after SDT. A significant amount of green fluorescence signal was observed on the surface of tumor cells in the HPNDs@EcN + US group compared to the weak fluorescent signal in the other groups, indicating that CRT was greatly exposed on the surface of tumor cells (Fig. 3A and B). Under ultrasound irradiation, the fluorescence intensity of HMGB1 in the nucleus of 4T1 cells in the HPNDs@EcN + US group was significantly lower than that in the control, US, and HPNDs@EcN groups, indicating that this nanodroplet bacterial complex exocytosed HMGB1 in the nucleus under the action of ultrasound (Fig. 3C and D).

### In vivo targeting ability of HPNDs@EcN

**Fig. 4:**
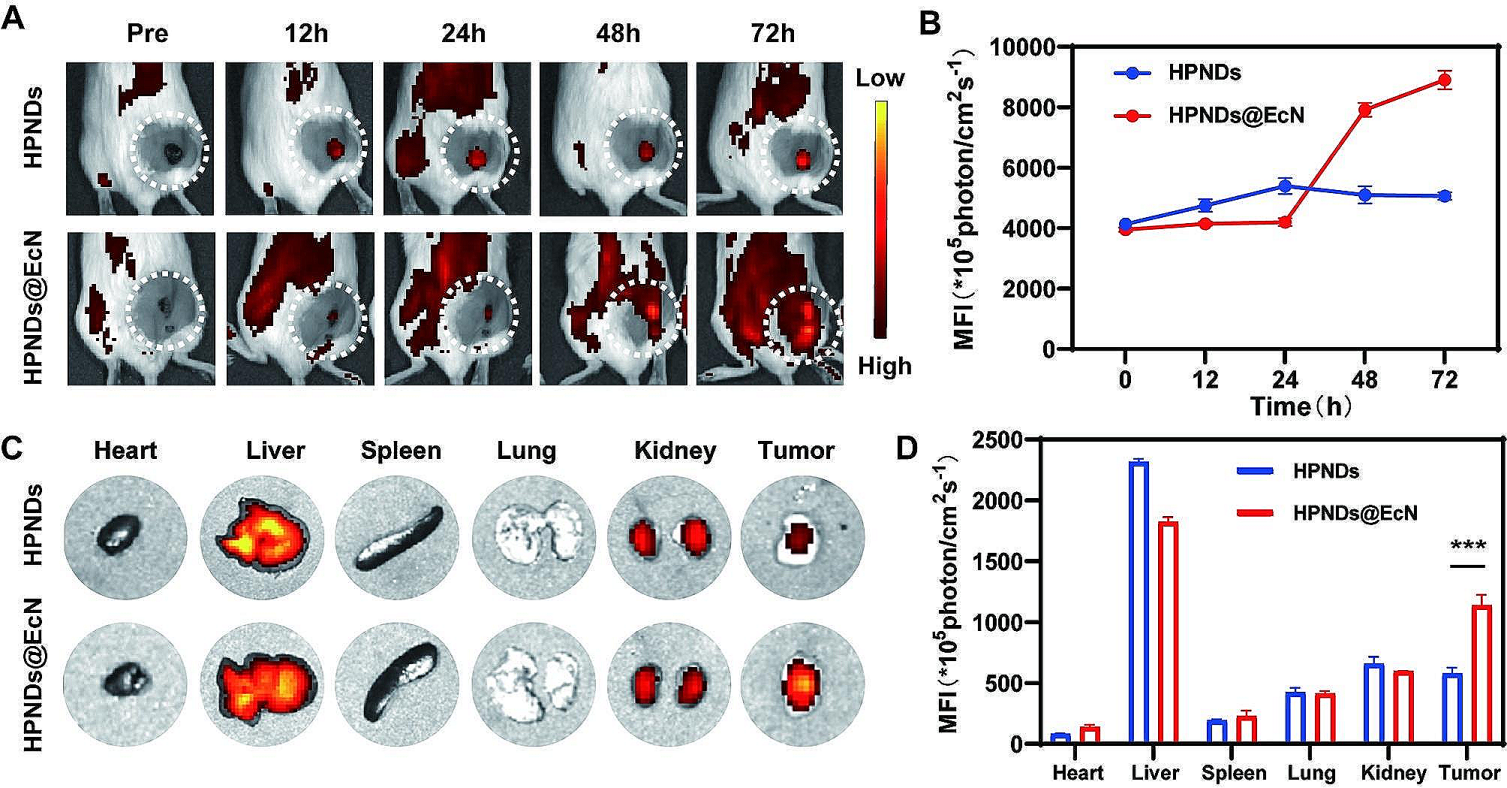
In vivo tumor targeting ability of HPNDs@EcN. (**A**-**B**) In vivo fluorescence imaging and fluorescence quantification in tumor-bearing mice after tail vein injection of HPNDs@EcN. (**C**-**D**) Ex vivo fluorescence imaging of various organs and quantitative analysis. (*** *p* < 0.001)

The distribution pattern of HPNDs@EcN in vivo after tail vein administration, especially the amount of local aggregation in the tumor, has a significant impact on the final therapeutic efficacy. To assess HPNDs-EcN ability to target tumor regions, DIR with promising fluorescence imaging characteristics was utilized to label bacteria without destroying the stability of HPNDs-EcN. The fluorescence signal in the tumor area was observed for 72 h after the injection of HPNDs and HPNDs@EcN, and it was found that the intensity of the fluorescence signal increased with time. When compared to HPNDs injection, the tumor area of mice injected with HPNDs@EcN had a higher fluorescence signal at 48 h, peaking at 72 h after injection (Fig. 4A and B). After 72 h, the heart, liver, spleen, lung, kidney and tumor were obtained for e*x vivo* fluorescence imaging, and it was found that there were strong fluorescence signals at both liver and tumors in both groups. Among them, the mice injected with HPNDs@EcN showed 2 times higher in signal intensity in the tumor region than those injected with HPNDs (Fig. 4C and D), which was consistent with the in vivo imaging data shown in Fig. 4A and B. These findings revealed that HPNDs@EcN have better tumor targeting ability than HPNDs alone.

To further confirm the targeting ability of number HPNDs@EcN to tumor region, the number of viable bacteria in each organ and tumor in vivo was determined at 24 h, 48 h and 72 h after the tail vein administration of HPNDs@EcN. An equal amounts of heart, liver, spleen, lung, kidney, and tumor tissues were grinded and underwent colonies counting to monitor the distribution pattern of HPNDs@EcN in vivo. As shown in Fig. S3, the number of viable bacteria in tumor tissues was much higher than that in heart, liver, spleen, lung, and kidney tissues from 24 to 72 h after the injection of HPNDs@EcN, and the number of colonies in tumor tissues tended to increase with time.

### In vivo anti-tumor efficiency of HPNDs@EcN

**Fig. 5:**
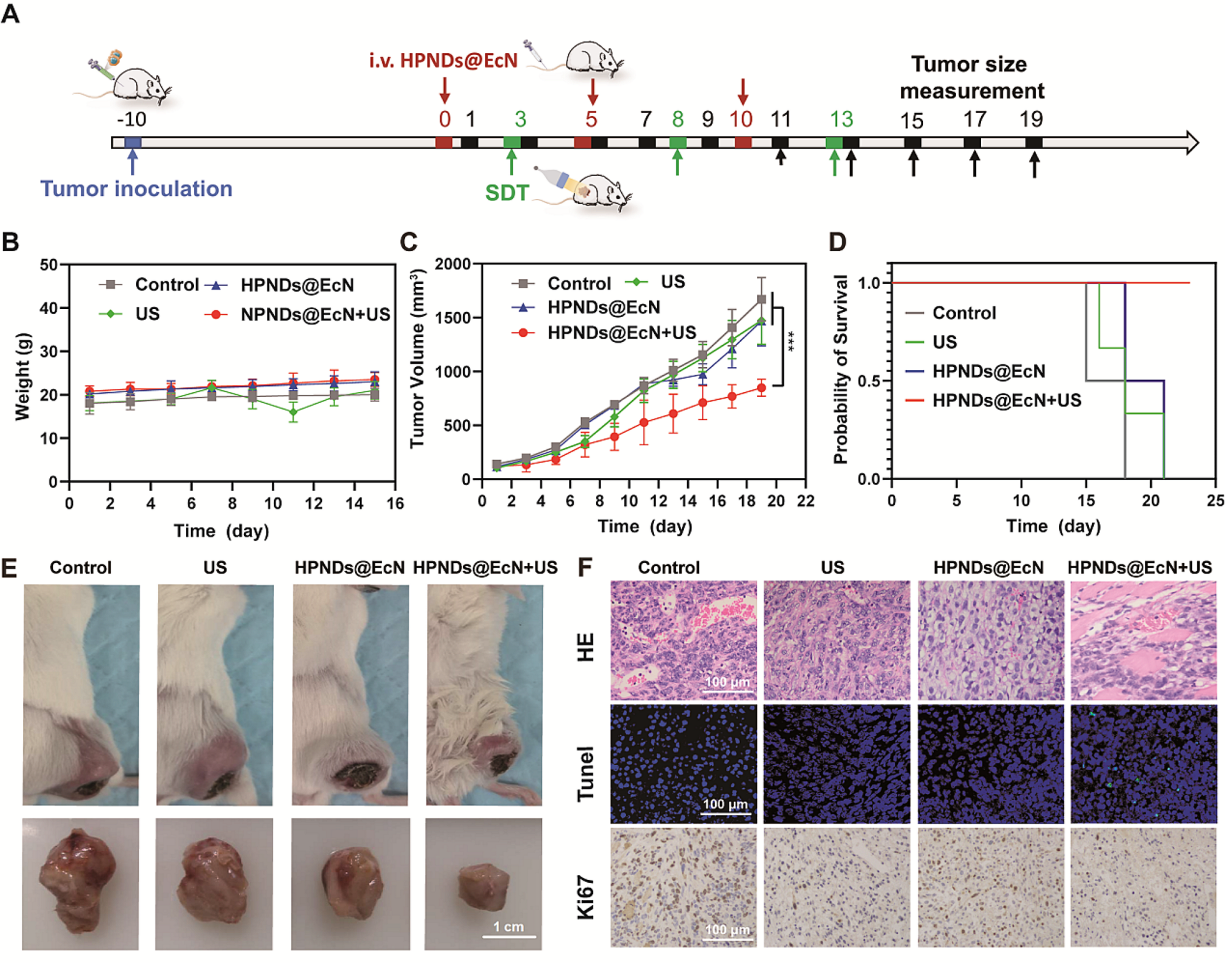
In vivo anti-tumor efficiency of HPNDs@EcN. (**A**) Schematic diagram of the treatment process of tumor-bearing mice. (**B**) Body weight curve of tumor-bearing mice. (**C**) Tumor volume growth curve of tumor-bearing mice. (**D**) Survival curve of tumor-bearing mice. (**E**) Tumor growth of each group of tumor-bearing mice after the end of treatment. (**F**) H&E staining, TUNEL immunofluorescence staining, Ki67 immunohistochemical staining of tumor sections from tumor-bearing mice after receiving different treatment regimens. (*** *p* < 0.001)

Mice with 4T1 mammary carcinomas were randomly divided into 4 groups: control group (PBS), US group, HPNDs@EcN group, and HPNDs@EcN + US group, respectively. PBS and HPNDs@EcN were injected on days 0, 5, and 10, respectively, and ultrasound irradiation was performed 72 h after injection to investigate the therapeutic effect of HPNDs@EcN on breast cancer by continuous tumor volume monitoring (Fig. 5A). Compared with the control, US, and HPNDs@EcN groups, the tumor growth rate in the HPNDs@EcN + US group was slower and the tumor volume was significantly lower than that of each other group, but there was no significant change in the body weight of the mice (Fig. 5B and C). Except for the HPNDs@EcN + US group, all other groups of mice died within 22 days of treatment, suggesting that the HPNDs@EcN + US group was able to significantly prolong the survival time of tumor-bearing mice (Fig. 5D). At the end of treatment, mice with subcutaneous tumors in situ were dissociated and the tumor volumes of each group were compared. Subcutaneous tumors were visible on the right side of the body in all groups, and the size of subcutaneous tumors in the HPNDs@EcN + US group was significantly smaller than that in the other groups (Fig. 5E).

The morphology of tumor tissue in each group was observed by H&E staining after the end of treatment (Fig. 5F). The experimental results showed that larger cavity formation was seen in the HPNDs@EcN + US group compared with the other groups. In the TUNEL staining results of tumor tissues of each group, the HPNDs@EcN + US group had relatively more green fluorescent signals, indicating that the maximum amount of cell apoptosis occurred in the HPNDs@EcN + US group compared with the other groups. Immunohistochemical staining results of Ki 67 showed that the positive rate of HPNDs@EcN + US was lower than that of the other groups, indicating that tumor cells in the HPNDs@EcN + US group had decreased proliferative capacity. Furthermore, H&E staining of the heart, liver, spleen, lung, and kidney showed no histopathological lesions, indicating the biocompatibility of HPNDs@EcN and the related treatment modalities (Fig. S4).

### In vivo anti-tumor metastasis and ICD measurements

**Fig. 6:**
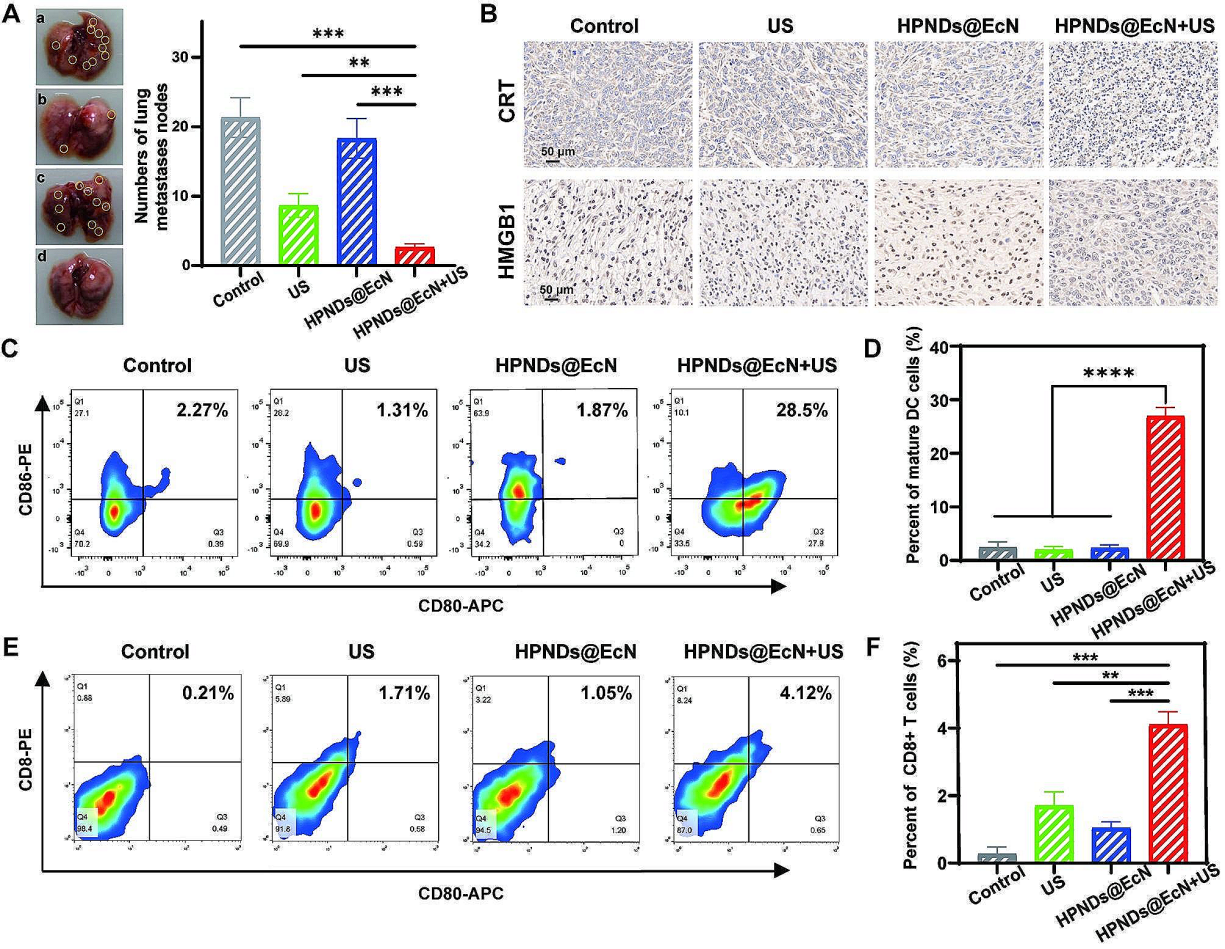
In vivo anti-tumor metastasis and ICD measurement. (**A**) Number of pulmonary metastases (a: Control; b: US; c: HPNDs@EcN; d, HPNDs@EcN + US). (**B**) Immunohistochemistry of CRT and HMGB1 in tumor tissue after various treatments. (**C**-**D**) Flow quantification of mature dendritic cells in tumor tissue after various treatments. (**E**, **F**) Flow quantification of infiltrated CD8 + T cells in tumor tissue after various treatments. (** *p* < 0.01, *** *p* < 0.001, **** *p* < 0.0001)

In the process of tumor development, metastases often accompany the occurrence of metastases, and the lung is a common organ with high tumor metastasis. Therefore, the inhibitory effect of HPNDs@EcN-mediated acoustic power therapy on tumor lung metastasis can be initially evaluated by comparing the number of lung metastases in each group. The mean number of metastatic tumor nodules in the lungs of the HPNDs@EcN + US group was significantly less than that of the other groups (Fig. 6A).

CRT and HMGB1 are key biomarker molecules for immunogenic cell death. To investigate the ability of HPNDs@EcN + US to induce ICD in tumor cells and promote the body’s tumor immune response, the exposure levels of intra-tumor cell CRT and HMGB1 were detected by immunohistochemistry. The experimental results showed that tumor cells in the HPNDs@EcN + US group had stronger CRT cell exposure and HMGB1 exocytosis than the control group (Fig. 6B). Theoretically, DC cells in vivo can be recruited to accumulate towards tumor tissue and deliver antigens to lymphocytes, promoting infiltration of immune cells such as lymphocytes. Mature DC cells can uptake, process, and present antigens, and also can activate T cells, which play a crucial role in immunotherapy. Therefore, flow assay evaluation of mature DC cells within tumor tissues showed that the HPNDs@EcN + US group exhibited a stronger ability to promote DC cell maturation. Compared to the control group, the proportion of mature DC cells in HPNDs@EcN group was about 12.5 times that in the control group (Fig. 6C and D). The quantitative assessment of the CD8 + type T cell predominance in tumor tissues was also performed, and the results showed that the HPNDs@EcN + US group exhibited good pro-CD8 + type T cell infiltration in tumor localization, further illustrating the ability of HPNDs@EcN + US to induce tumor immune responses in the body (Fig. 6E and F).

## Discussion

In this study, we designed a bacterial nanocomposite HPNDs@EcN for SDT. One of the important influences of SDT-induced ICD is the triggering of ROS production by sonosensitizers [34]. As we all known, ROS can induce tumor cell death by changing the cytoskeleton and blocking the cell cycle [35]. However, many studies have shown that excess ROS can also cause irreversible damage to normal cells, which can lead to cell death [36]. Therefore, to achieve the maximum therapeutic effect and avoid damage to normal tissues, it is the most ideal treatment method to gather the sonosensitizers to centrally generate ROS at the tumor site. Nonetheless, due to the presence of a complex TME [37, 38], conventional sonosensitizers of inorganic materials cannot accumulate well locally in tumors. Nanoparticle carried sonosentiziters is a strategy that can achieve controlled release [39]. But there are also limited by the specific characteristics of the TME, such as lack oxygen, low pH, lack of blood vessels and so on. That is why we selected probiotics as the carries of sonosensitizer-loaded nanoparticle.

Bacterial nanocomposites have shown great potential in cancer treatment. Among them, probiotics are a representative biological carrier that can accumulate in solid tumors due to the chemotaxis of their biochemicals in the TME, and are less toxic in vivo to achieve long-term antitumor effects [40]. Building on the fact that our team has already developed a SonoBacteriaBot, which offers significant benefits in terms of real-time monitoring and control of drug release [31], we continue to leverage the targeting effect of probiotics to provide a promising basis for the aggregation of sonosensitizers. We used DIR to label the bacteria and perform continuous monitoring of their distribution in mice, and ex vivo imaging of mouse organs showed that HPNDs@EcN have higher fluorescence intensity in tumors than HPNDs only (Fig. 4C and D). These results indicated that HPNDs@EcN had good tumor targeting, which provided a good condition for SDT-induced ICD and greatly reduced the damage to normal tissues and organs.

In addition to excellent tumor targeting, the HPNDs@EcN also had the effect of local treatment of tumor for a long time which is a prerequisite for an effective ICD. In our studies, when HPNDs@EcN was injected through the mouse tail vein, the tumor site maintained a high fluorescence signal at 48–72 h compared with injected HPNDs and was slowly strengthened by about 25% at 72 h post-injection compared to 48 h basis (Fig. 4A and B). Based on this premise, we performed three ultrasound-mediated treatments in the animal experimental phase and achieved good therapeutic results in the subsequent period.

Back to the essence of cancer treatment, it is estimated that each cell undergoes more than 20,000 events of DNA damage each day that can be repaired. Even some cells that are unable to repair or acquire malignancy are often recognized and killed by the host immune surveillance system [41]. Therefore, cancer develops when the host immune system fails to eliminate these mutated. Because of this, immune checkpoint blockade (ICB) therapies have been widely used and achieved certain clinical benefits, but some patients eventually end in relapse and progress [42]. In our study, it was proved that HPNDs@EcN based SDT can not only effectively inhibit the growth of local tumors, but prevent the spread of tumors to lung tissues. Such a result of remote inhibition may be explained by the Effector Immune Cell Deployment (EICD) principle of Academician Erwei Song,  who has recently proposed the concept in a featured review published in Trends in Immunology [43]. Unfortunately in this study, we only made a preliminary exploration of the mechanism of SDT-mediated ICD, and preliminarily proved that SDT can induce the exposure of CRT on the surface of tumor cells in vivo and in vitro, but did not comprehensive analysis of not just the abundance of T cells infiltration, but also the balance of tumor-infiltrating T lymphocytes repertoire, the percentages of “bystander” T cells and the specific subsets of exhausted T cells [41]. In the follow-up study, relevant experiments should be supplemented to fully explore the specific mechanism of SDT enhancement of ICD and remote inhibition.

### Electronic supplementary material

Below is the link to the electronic supplementary material.

Supplementary Material 1

## Data Availability

The datasets used and/or analysed during the current study are available from the corresponding author on reasonable request.
