# Supplementary material for: Bacteria-driven nanosonosensitizer delivery system for enhanced breast cancer treatment through sonodynamic therapy-induced immunogenic cell death
Source: J Nanobiotechnology. 2024 Apr 12;22:167. doi: 10.1186/s12951-024-02437-0 (PMC11010413; doi:10.1186/s12951-024-02437-0)
Supplement: Supplementary file 1 — Supplementary Material 1 [file 12951_2024_2437_MOESM1_ESM.docx]

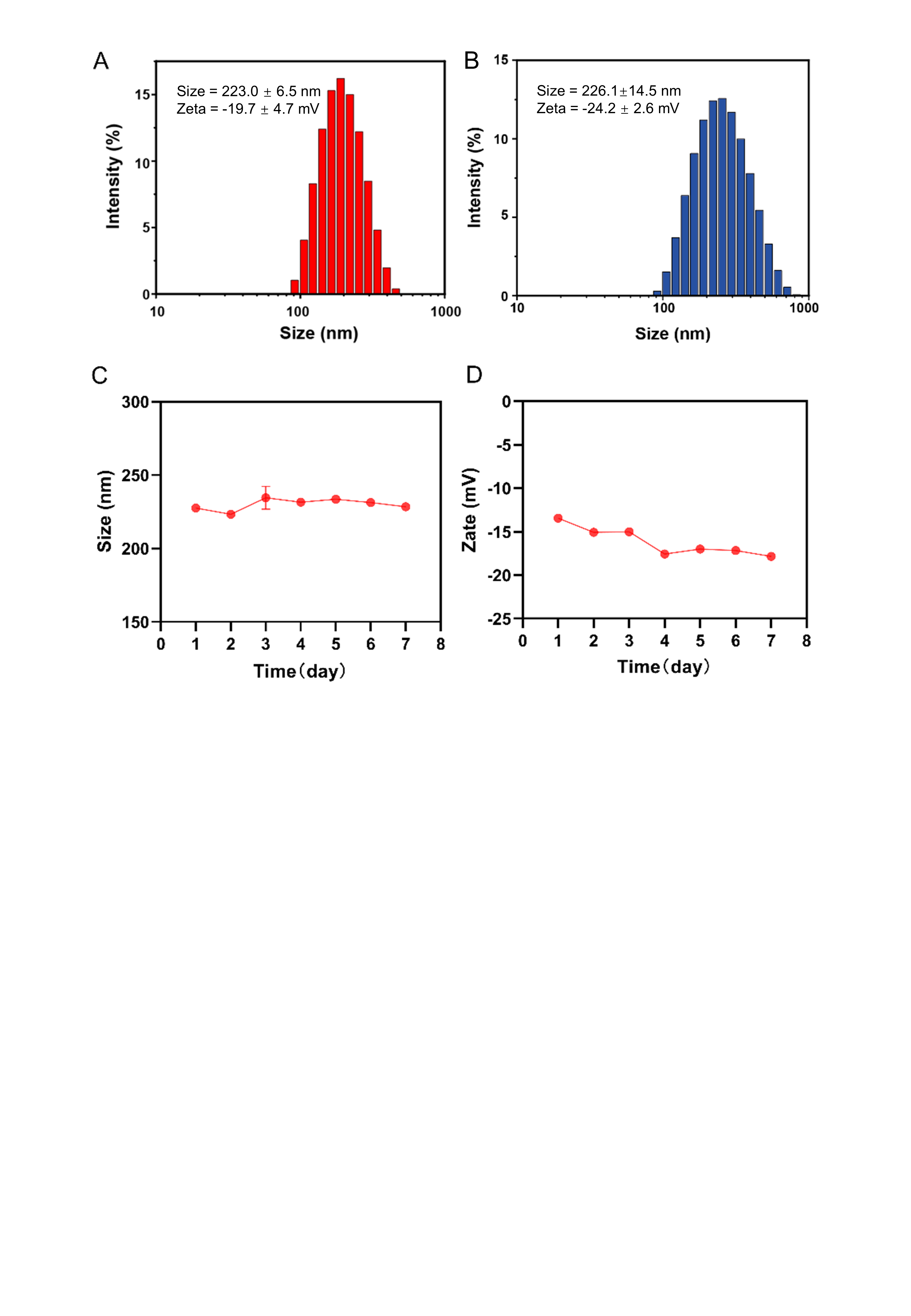


**Figure S1:** Particle size distribution, zeta potential, and stability of HPNDs. (A) Particle size distribution and zeta potential of HPNDs. (B) Particle size distribution of HPNDs after ultrasound irradiation. (C) Particle size distribution of HPNDs in PBS at different time points. (D) Zeta potential of HPNDs in PBS at different time points.


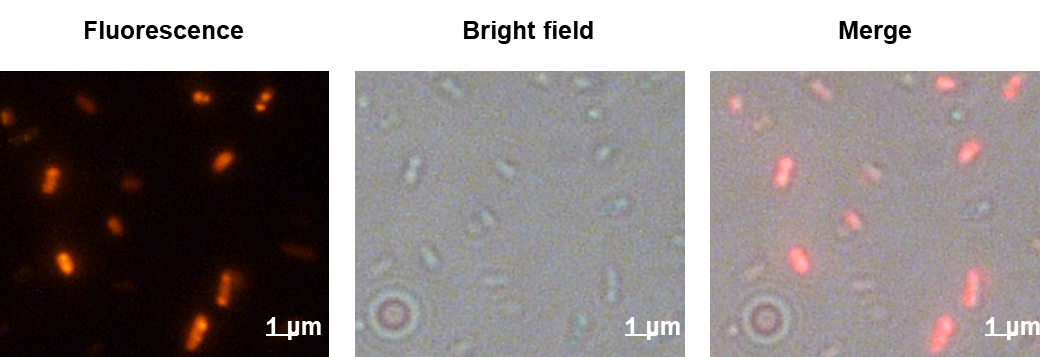


**Figure S2:** Fluorescence image of HPNDs@EcN. (Red: HMME)


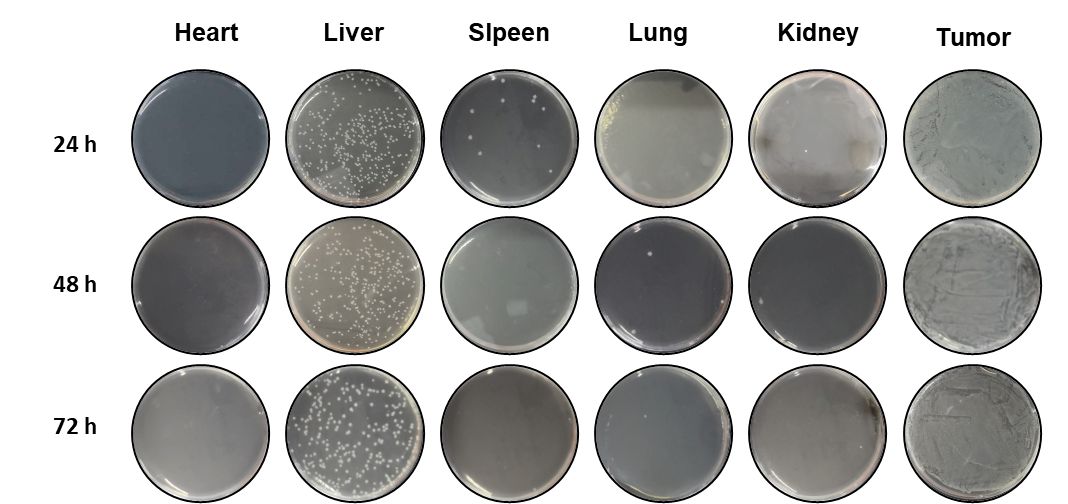


**Figure S3:** Smear plate of each organ in tumor-bearing mice after 24 h, 48 h, and 72 h tail vein injection of HPNDs@EcN. Accumulation of EcN in tumor, heart, liver, spleen, lung, and kidney was detected by colony counting.


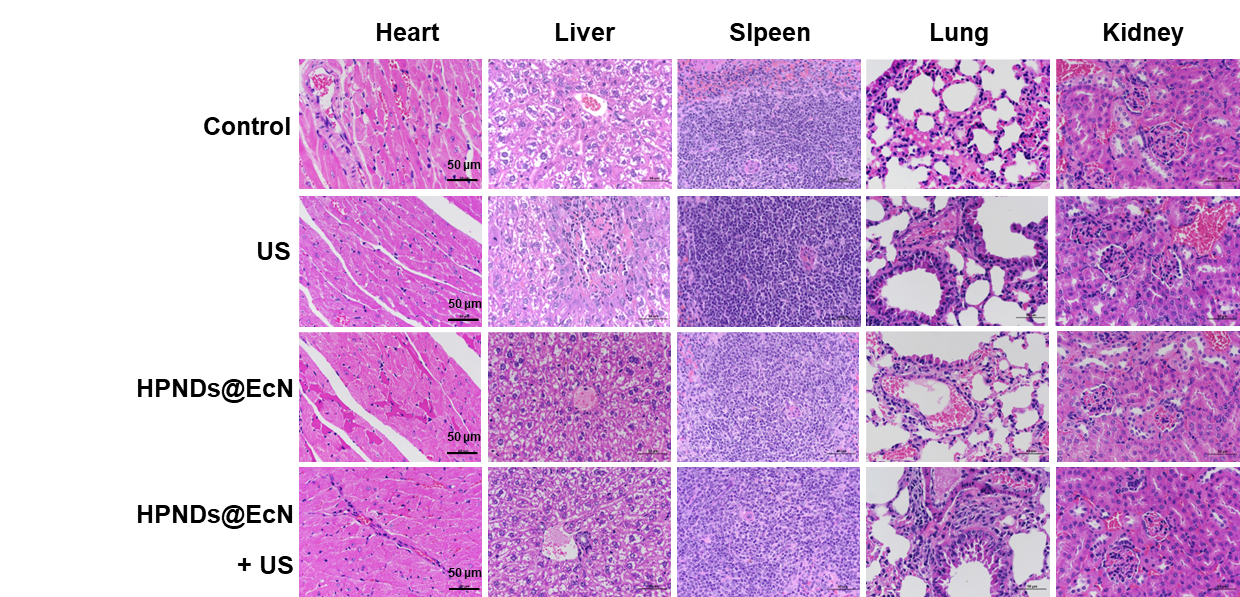


**Figure S4:** *In vivo* biosafety evaluation of HPNDs@EcN by H&E staining of major organs of mice after various treatments. (Scale bar: 50 μm)
